# Atlas of Clinical PET in Oncology

**DOI:** 10.1054/bjoc.2000.1478

**Published:** 2000-11-22

**Authors:** P Price

**Affiliations:** Radiation Oncology, Manchester University & Head of PET Oncology Group, MRC Cyclotron Unit Imperial College School of Medicine Hammersmith Hospital, Du Cane Road, London, UK


*British Journal of Cancer* (2000) **83**(11), 1581
© 2000 Cancer Research Campaign
doi: 10.1054/ bjoc.2000.1478, available online at http://www.idealibrary.com on IDEAL®

http://www.bjcancer.com

### Book review

### Atlas of Clinical PET in Oncology

H Bender, H Palmedo, H-J Diersack and PE Valk (eds)
Published by Springer

If you are an oncologist or a nuclear medicine physician using clinical FDG PET for the diagnosis of cancer, you should have a copy of this book. It is a beautifully presented atlas of whole body and transaxial PET images in various oncological situations. It will certainly make an excellent reference text book as well as being a useful learning experience for those not familiar with diagnostic PET in oncology.

This is a multi-authored book from many of the international leaders in the field. Following the introduction of whole-body PET over the last few years, there has been an explosion in diagnostic PET in oncology. This therefore is a very timely book giving examples of all the main tumour types and their appearance with FDG PET.

The book is well laid out with very clear illustrations. The atlas compares PET with CT and MRI and other imaging which is extremely useful. Also as this is a multi-author work and therefore multi-institution there is a useful variation in the whole-body images displayed.

The book is divided into a very neat introduction and then an excellent chapter on normal appearances of FDG, very important for the beginner. The following chapters are on individual tumour sites. The chapters individually give an introduction and then go through case studies with very clear and tight summaries of the cases with illustrations. What is perhaps the most useful part of the book is the false positives that are included and some useful 'pit falls' which the authors kindly share with us. As it takes many years to acquire the knowledge for good interpretation of whole-body PET visually, this is a vital text book for many. It is well referenced and provides most of what one needs to know.

Readers, however, should take note of the preface from the editors which states 'in the editors' experience it is necessarily in this context to correlate PET images with CT, MRI and histopathology, not only in order to achieve the best available information about the patients but also to learn about the advantages and limitations of PET'.

*Prof. P. Price*
*Professor of Radiation Oncology, Manchester University*
*& Head of PET Oncology Group,*
*MRC Cyclotron Unit*
*Imperial College School of Medicine*
*Hammersmith Hospital*
*Du Cane Road*
*London, UK*

